# Rheumatoid Arthritis and the Cervical Spine: A Review on the Role of Surgery

**DOI:** 10.1155/2015/252456

**Published:** 2015-08-17

**Authors:** John L. Gillick, John Wainwright, Kaushik Das

**Affiliations:** Department of Neurosurgery, NY Medical College, 19 Skyline Drive, Hawthorne, NY 10532, USA

## Abstract

Rheumatoid arthritis (RA) is a chronic systemic inflammatory disease affecting a significant percentage of the population. The cervical spine is often affected in this disease and can present in the form of atlantoaxial instability (AAI), cranial settling (CS), or subaxial subluxation (SAS). Patients may present with symptoms and disability secondary to these entities but may also be neurologically intact. Cervical spine involvement in RA can pose a challenge to the clinician and the appropriate role of surgical intervention is controversial. The aim of this paper is to describe the pathology, pathophysiology, clinical manifestations, and diagnostic evaluation of rheumatoid arthritis in the cervical spine in order to provide a better understanding of the indications and options for surgery. Both the medical and surgical treatment options for RA have improved, so has the prognosis of the cervical spine disease. With the advent of disease modifying antirheumatic drugs (DMARDs), fewer patients are presenting with cervical spine manifestations of RA; however, those that do, now have improved surgical techniques available to them. We hope that, by reading this paper, the clinician is able to better evaluate patients with RA in the cervical spine and determine in which patients surgery is indicated.

## 1. Introduction

Rheumatoid arthritis (RA) is a chronic systemic inflammatory disease that primarily affects bones, synovial joints, and ligaments but can also involve nearly every organ system. RA primarily affects the peripheral joints; however it can also have profound systemic effects on the cardiovascular, pulmonary, and hematologic systems [1–3]. RA affects an estimated 1-2% of the world's adult population. In the United States an estimated 1.5 million adults are affected, and there are approximately 41 new diagnoses of RA being made per 100,000 individuals over the age of 18 every year [1, 4]. While the most prominent effects of RA are observed in small peripheral joints, the second most commonly involved region is the cervical spine [2, 3, 5, 6]. First described in 1890 by Garrod, who noted cervical spine involvement in 178 (35%) of 500 patients with RA, more recent estimates suggest that upwards of 80% of patients with RA have radiographic cervical spine involvement, some as early as within 2 years of initial diagnosis with RA [1, 2, 5, 7]. Chronic inflammation of the cervical spine initially leads to proliferation of fibrovascular tissue and pannus formation resulting in bony erosion and ligamentous laxity. This cascade can lead to cervical spinal instability in the form of atlantoaxial instability (AAI), cranial settling (CS), and subaxial subluxation (SAS) or a combination of the three [1–3, 5, 7]. Additionally, RA can cause an inflammatory discitis and atraumatic odontoid erosion or fracture [7]. Cervical spine involvement is of particular importance because, when left untreated, it can lead to significant neurologic morbidity, worsening quality of life, and possibly sudden death from stroke, obstructive hydrocephalus, or cardiac arrest [1, 5, 8]. Although the medical treatment of RA has been improved with disease-modifying antirheumatic drugs (DMARDs) and biologic agents (BAs) that have been shown to decrease the incidence of initial cervical spine involvement, these agents are unable to prevent progression of cervical disease once it occurs in contrast to their success in treating peripheral joint manifestations [2, 5, 7]. When cervical spine involvement becomes symptomatic, surgical stabilization should be considered as it has been shown to delay and sometimes prevent progression of disease and improve functional status in certain patients [1–3, 5, 7]. Because of the severe and potentially deadly complications of cervical spine disease in RA, its early diagnosis and treatment should be a priority in patients with RA. In this review, we will discuss the epidemiology of cervical spine disease in RA, provide a brief overview of the pertinent pathophysiology, and discuss the clinical manifestations, diagnostic evaluation, and indications for surgery while providing an overview of modern surgical approaches to cervical instability as well as outcomes.

## 2. Epidemiology of Cervical Spine Involvement

The epidemiology of cervical spine involvement in RA is difficult to describe due to wide variation in the available literature, likely owing to differences in study populations and design. In addition, much of the work describing the natural history of cervical spine involvement in RA was conducted prior to the development of DMARDs and BAs, making it less useful in the context of modern medical therapy [7].

In patients with RA, the prevalence of cervical involvement has been reported to range from 43 to 86% [1, 2]. One of the earliest indicators of cervical spine involvement in RA is neck pain, and as many as 40 to 88% of RA patients report complaints of this symptom [1]. In a study of 1,120 Korean RA patients who presented to a rheumatology clinic with neck pain, 320 (28.6%) had cervical spine involvement on initial evaluation [5, 10]. Several studies have also concluded that the presence or development of peripheral joint erosions, DMARD failure, prolonged corticosteroid use, and higher disease activity (as evidenced by elevated erythrocyte sedimentation rate or C-reactive protein level) were significant risk factors for the development or presence of cervical spine involvement [2, 5, 11–14].

As mentioned above, cervical spine involvement in RA can occur early in the course of the disease. A recent prospective study conducted by Yurube et al. found that out of 140 RA patients who were without cervical spine involvement at baseline, 61 (43.6%) developed instability, primarily AAI, after a minimum of 5 years of follow-up [12]. The development of AAI is significant as patients are likely to have progression of their cervical instability with worsening of their AAI or development of CS, which significantly increases the risk of developing neurologic deficit [1, 2]. In a different study, Yurube and coworkers reported that over 5 years of follow-up, 22.8% of patients with AAI at baseline progressed to CS and 33.3% of patients developed “severe” instabilities that were likely to cause spinal cord compression [13]. Other authors have reported progression rates as high as 80 to 87% over 6 to 10 years [18, 19]. As cervical instability progresses, SAS has been observed to occur in 10 to 20% of those patients [2]. Based on available literature, it can be reasonably concluded that cervical spinal instability begins with AAI and as it progresses, CS and SAS develop.

The development of cervical spinal instability in RA patients can have dire consequences. While it is estimated that 7 to 34% of patients with radiographic cervical instability will report neurologic deficits at the time of diagnosis, 36% of patients with preexisting instability will have neurologic progression. Neurologic progression contributes to mortality in RA patients, with reported mortality rates as high as 50% in the first year of developing myelopathy [1]. In a study of 21 RA patients with cervical instability and myelopathy who refused surgery, 16 (76%) had further neurologic progression, all patients were bedridden within three years of developing myelopathy, and the cumulative probability of survival was 0% in the first 7 years after developing myelopathy [2, 20]. Riise et al. reported a mortality rate in RA patients with cervical instability 8 times higher than those without instability [21]. Interestingly, they also report that all the patients with cervical instability who died had high RA disease activity [21]. It is also important to note that once cervical instability has progressed to CS, the risk of sudden death is significantly increased [22].

## 3. Anatomy

The C1-C2 complex is responsible for 60° of axial rotation [23]. The ligaments and articulations of the occipitoatlantoaxial complex control mobility and restriction in movement. The ring of C1 articulates with the base of the skull via the occipital condyles and is restrained by the tectorial membrane. Atlas (C1) is also connected to the skull via the anterior atlantooccipital membrane, which connects the anterior arch of C1 to the anterior margin of the foramen magnum. Additionally, the posterior arch of C1 is connected to the posterior margin of the foramen magnum via the posterior atlantooccipital membrane.

**Figure 1:**
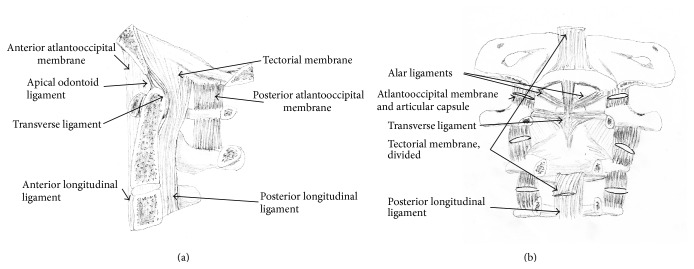
(a) Sagittal view of occipitocervical complex. (b) Posterior view of occipitocervical complex. (Reprinted with permission [9]. Promotional and commercial use of the material in print, digital, or mobile device format is prohibited without the permission from the publisher Lippincott Williams & Wilkins. Please contact journalpermissions@lww.com for further information.)

Axis (C2) has several articulations with atlas (C1) to reinforce the occipitoatlantoaxial complex. The anterior arch of C1 articulates with the odontoid process of C2 in a synovial joint that is constrained by the transverse ligament, which holds the dens to the anterior arch of C1 via a strap-like mechanism, and prevents anterior translation of C1 relative to C2. This anatomy is susceptible to damage from RA, resulting in neurologic compression and craniocervical instability. The occipitoatlantoaxial complex is depicted in Figure 1.

## 4. Pathophysiology

Involvement of the cervical spine in RA occurs due to progression of synovial inflammation. The pathophysiology involved in this process involves a complex interaction between genetic, environmental, and immunologic factors. The precise etiology is unknown; however, a more detailed molecular pathway is emerging. Environmental factors combine with genetic predisposition (specifically those with HLA-DR4 and DR-1) activating antigen-presenting cells (APCs), which in turn stimulate CD4+ T cells [9]. These activated CD4+ T cells then activate B-lymphocytes to produce plasma cells, which secrete autoantibodies (rheumatoid factor (RF) and anti-cyclic citrullinated peptides (anti-CCPs)), promoting further inflammation [24]. In addition, activated T cells stimulate macrophages, which release proinflammatory cytokines such as TNF-*α*, IL-1, and IL6. In particular, TNF-*α* plays a crucial role in pathogenesis as it promotes leukocyte influx and activates fibroblasts, which then secrete matrix metalloproteinases (MMPs) causing breakdown of articular cartilage [24]. TNF-*α* also increases the expression of receptor activator of nuclear factor *κ*B ligand (RANKL), which activates osteoclast differentiation and bone resorption [24]. These processes cause bone and cartilage degradation via an immune-modulated mechanism. If allowed to progress, these processes can result in spinal instability from damage to the ligamentous complexes resulting in laxity, mechanical neural compression due to overgrowth (as observed with pannus formation), or impaired blood supply to the spinal cord [25].

## 5. Clinical Manifestations

The clinical manifestations of cervical disease in RA are varied and difficult to interpret in the setting of the joint arthropathy, muscle wasting, decreased range of motion, compressive neuropathy, and poor functional status of many patients [2]. It is important to note that the incidence of asymptomatic cervical involvement in RA is high with reports of 33 to 50% of patients having no symptoms, and thus heightened awareness of the frequency of cervical involvement is paramount in the early detection of the beginning stages of the disease even in the absence of symptoms [14, 19, 22]. There are several findings that should prompt further investigation and raise suspicion for cervical involvement. Neck pain, specifically pain at the craniocervical junction, is one of the most common presentations, in one report occurring in 69% of patients with cervical instability [1, 7, 10, 14]. Occipital headache is also a common complaint, present in 60% of AAI and 90 to 100% of CS, and can be attributed to compression of the greater and lesser occipital nerves as they pass between C1 and C2 [1, 7, 26]. Also, compression of the greater auricular nerve can result in ear or mastoid pain [1]. In addition, careful history taking patients with AAI may describe crepitation or a sensation of their head “falling forward” with flexion that may be reproduced with appropriate physical exam maneuvers revealing a palpable “clunking” [1, 2, 7, 16].

It is crucial not to miss signs of myelopathy given the increased morbidity and mortality associated with the onset of neurologic deficit in patients with RA. Such signs can include muscle atrophy, weakness, limb paresthesias, bowel and/or bladder disturbance, hyperreflexia, spasticity, increased Hoffman's reflex, abnormal plantar reflex signs, abnormal abdominal reflexes, and loss of proprioception [1, 2, 7]. Patients with compression of the upper spinal cord and cervicomedullary junction report the presence of Lhermitte's sign, a shooting electric sensation that runs down the back, on neck flexion often accompanied by the above-mentioned “clunk” [1, 2]. Patients with more severe compression of the cervicomedullary junction can have abnormalities of the lower cranial nerves such as dysphagia from compression of the vagus and glossopharyngeal nerves, dysarthria from compression of the hypoglossal nerve, loss of facial sensation or facial pain from compression of the nucleus of the spinal trigeminal tract, syringomyelia, and even locked-in syndrome or sudden death [1–3]. Cranial nerve involvement has been reported in up to 20% of patients; however, work by Rogers et al. did suggest that cranial nerve involvement and brain stem dysfunction may be due to the comorbidities associated with RA and not cervical instability [2, 7, 27]. Signs of vertebrobasilar insufficiency such as tinnitus, vertigo, visual disturbance, and dysphagia can also occur and may be due to mechanical compression [1, 3]. Repeated vertebrobasilar thromboembolic events have also been reported in patients with severe cervical instability causing kinking of the vertebral arteries [8].

Patients with debilitating lower extremity arthritis seem to be especially susceptible to cervical spine involvement. In 101 rheumatoid patients who had undergone a lower limb arthroplasty, 82 were later found to have cervical spine instabilities. Furthermore, patients with AAI, CS, and SAS were found to have had more joint arthroplasties at final follow-up compared to those with less severe cervical involvement [28]. In addition, associations have been described between lower limb joint involvement and cervical spine disease. In fact, Imagama and colleagues found that severe large joint disease (disease of shoulders, elbows, hips, and knees) expressed as a “large joint index” correlated significantly with AAI, CS, and PADI. These authors concluded that involvement of the large joints might serve as a predictor of cervical spine instability [29].

The systemic effects of RA including the involvement of the peripheral joints, compressive neuropathies, and myelopathies make it difficult to use traditional neurologic grading systems. Several grading systems have been developed to classify the functional status of patients with RA and severity of myelopathy. The Ranawat Classification of Rheumatoid Myelopathy is one of the most commonly used systems (Table 1) [1–3, 15]. Another system is the American Rheumatologic Association Classification of Global Functional Status in Rheumatoid Arthritis (Table 2) [2, 17, 30]. These classifications are important because there are strong correlations with worsening morbidity and mortality with increasing Ranawat class [2]. In addition, these grading scales are useful in evaluating potential postoperative outcomes as there were increasing complications and morbidity with increasing class; however the majority of patients improved by one class postoperatively [1, 2, 31, 32].

## 6. Diagnostic Evaluation

**Figure 2:**
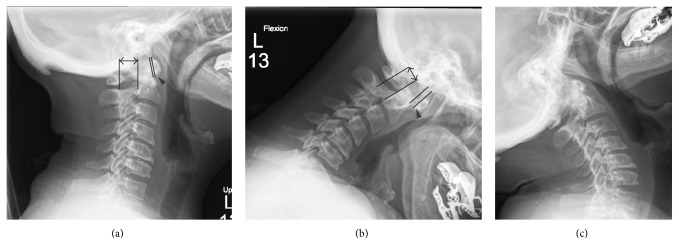
Lateral radiographs of a patient with atlantoaxial instability. In the neutral view the AADI (*arrowhead)* is 1 mm and the PADI (*double arrow)* is 20 mm (a). In flexion the AADI increases to 7 mm and the PADI decreases to 13 mm (b). In extension the AADI and PADI reduce to their neutral measures (c).

Given the high prevalence of asymptomatic cervical instability in RA patients, understanding the appropriate diagnostic evaluation is crucial to early detection. In the majority of patients without significant symptoms of cervical instability, plain radiographs consisting of standard anterior/posterior, lateral, and open mouth views in addition to dynamic lateral flexion/extension views are an appropriate initial evaluation as they are easy to obtain and inexpensive [1, 2, 7]. The flexion/extension views are critical as the standard static lateral projections have been reported to miss detection of AAI, underestimate its severity, and poorly evaluate stability [33]. When evaluating plain radiographs for cervical instability, several measurements can be made to assess for the presence and severity of disease. In order to evaluate for AAI, the anterior atlantodental interval (AADI) and the posterior atlantodental interval (PADI) can be measured. The AADI is the distance from the posterior margin of the anterior arch of C1 to the anterior margin of the dens measured along the transverse axis of C1 which in normal adults is less than 3 mm. AAI is defined as an AADI that is greater than 3 mm and not fixed with flexion and extension as it generally increases with flexion and may reduce with extension (Figure 2) [1–3]. Various cutoffs between 6 and 10 mm for maximum AADI have been suggested as indications for surgery [1–3, 7]. A limitation to the use of the AADI occurs in patients who have developed CS. Due to the conical shape of the dens, CS can result in a decrease in the AADI, which may become fixed, resulting in a pseudostabilization when in fact the patient has significant disease [2, 32, 34]. Due to the limitations of the AADI, PADI has been found to be a more reliable indicator of the potential for neurologic compromise [1, 2, 35]. This value is obtained by measuring from the posterior margin of the dens to the anterior margin of the posterior arch of C1 (Figure 2). Values for PADI less that 13 or 14 mm have been suggested as indications for surgery [3, 7].

**Figure 3:**
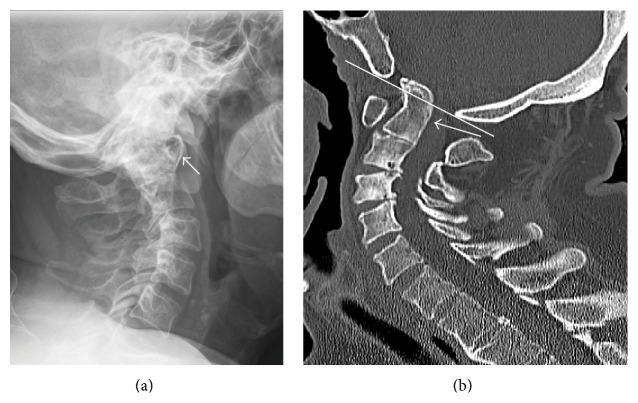
Lateral radiograph of patient with severe cranial settling. Of note, the settling is so severe that the dens is not identifiable due to overlying mastoid air cells and skull base,* arrow* identifying the anterior arch of C1 (a). Sagittal reconstructions of computed tomography of the cervical spine in the same patient. Note the anterior arch of C1 is at Station III and the dens (*arrow*) projects through the inferior margin (*line*) of the foramen magnum (b).

**Figure 4:**
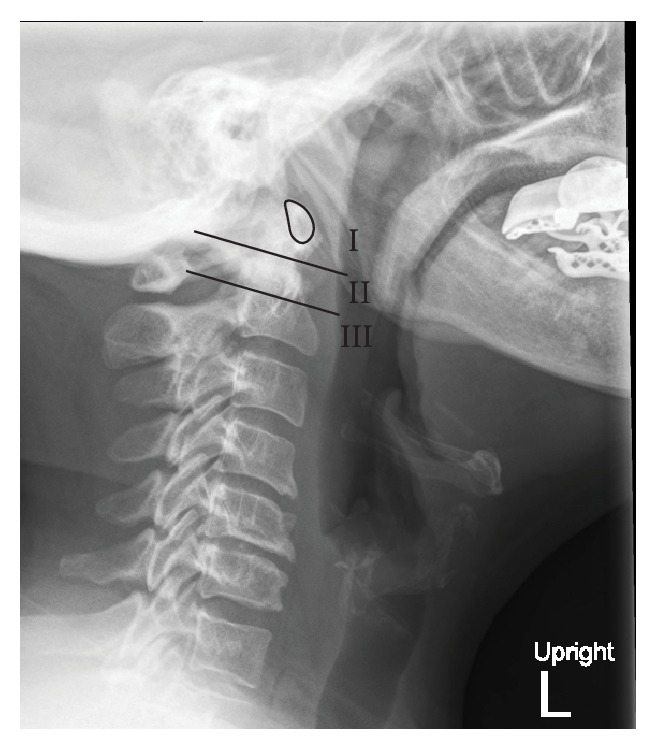
Clark station in the sagittal plane, divided into three equal parts (stations), and determined by the level at which the anterior arch of C1 (*outlined*) falls.

**Figure 5:**
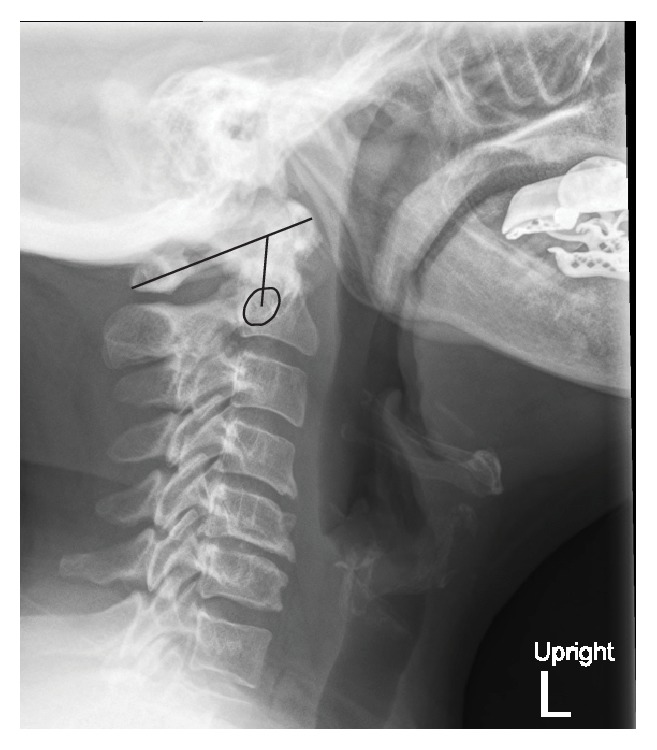
The Ranawat Criterion is the distance between the center of the C2 pedicle and the transverse axis of C1 measured along the axis of the odontoid process.

**Figure 6:**
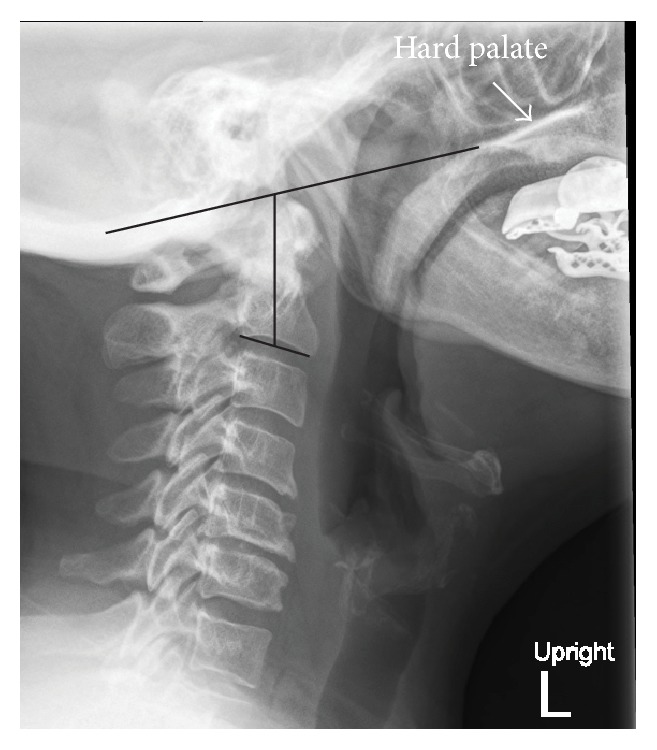
The Redlund-Johnell Criterion is the distance between the inferior margin of C2 vertebral body and a line drawn from the posterior tip of the hard palate (white arrow) to the caudal cortical margin of the occiput (McGregor Line).

There have been numerous measures proposed to evaluate radiographs for the presence and severity of CS; however these approaches have proven to be difficult to reproduce and as disease progresses, difficulty in visualizing landmarks complicates their use (Figure 3) [1, 2, 7, 36]. Based on the work by Riew et al., the presence of CS is best evaluated using a combination of the Clark station, Ranawat criterion, and the Redlund-Johnell criterion (Table 3) (Figures 4, 5, and 6). When at least one of these measures is positive, the sensitivity for detecting CS is 94% with a negative predictive value of 91%. However, this combination only has a positive predictive value of 56% meaning a large number of patients would be diagnosed as potentially having CS in the absence of disease and therefore magnetic resonance imaging (MRI) or computed tomography (CT) is recommended [36]. When considering the high morbidity associated with CS, this high false-positive rate may be considered acceptable.

Subaxial subluxation (SAS) is a result of inflammatory changes in the intervertebral discs, uncovertebral joints, and spinal ligaments causing loss of translational stability between vertebral bodies and intervertebral height loss. Traditional methods of diagnosis relied on measuring the amount of listhesis between adjacent vertebrae with SAS being present when there is 3.5 to 4 mm of listhesis between vertebrae [1, 2, 7]. More recently, the spinal canal diameter has been used as a measure for SAS with significant SAS being defined when the canal diameter is less than 13-14 mm [1, 2, 7].

One limitation of plain radiographs is that they can only evaluate bony structures and do not demonstrate the impact of retroodontoid pannus formation on the space available for neural structures. Therefore in patients with negative radiographs but symptoms suggestive of cervical instability or in patients with neurologic deficits, the use of advanced imaging such as CT and MRI is warranted to detect these changes [1, 5]. In addition to information about soft tissue compression, contrast enhanced MRI can allow for the early detection of cervical involvement prior to the development of erosive changes in bony structures, with the detection of enhancement of periodontoid synovial spaces, indicative of inflammatory synovitis, and marrow edema as early as three months from the initial diagnosis of early RA [37, 38].

## 7. Surgical Approaches

If left untreated, AAI can lead to poor clinical outcomes, morbidity, and possibly even death. In a case series of 21 patients, all refusing surgery, Sunahara and colleagues found that no patient showed any sign of improvement. In addition, 16 (76%) showed deterioration at follow-up. Interestingly, the probability of survival at 7 years was 0% following the onset of myelopathy [20]. This underscores the importance of timely treatment. In addition, it is postulated that untreated AAI can result in upward migration of the dens and CS due to the incompetence of the C1 lateral masses and decreased distance between the odontoid process and cranial cavity [2, 39]. In a case series by Grob, 20 patients were treated with atlantoaxial fusion for AAI on the basis of unsuccessful adequate conservative treatment. At 5-year follow-up, no patients showed progression to vertical cranial migration, suggesting a possible prophylactic role of atlantoaxial fusion [40].

**Figure 7:**
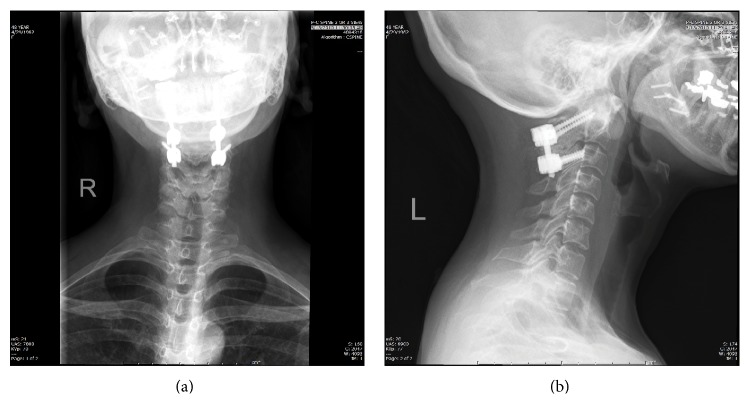
AP (a) and lateral (b) X-rays depicting a C1-2 fusion using C1 lateral mass and C2 pars screws.

**Figure 8:**
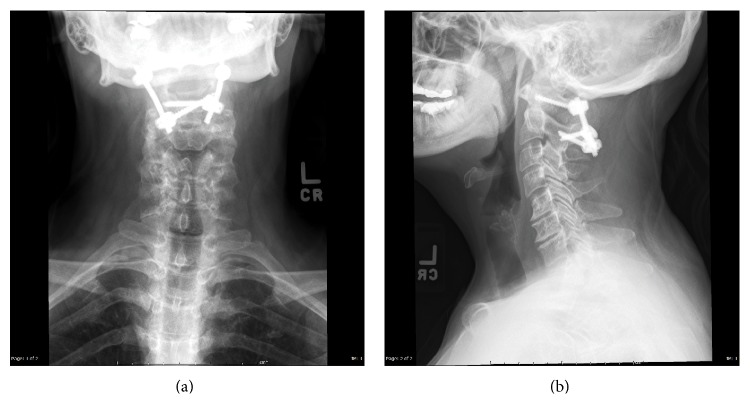
AP (a) and lateral (b) X-rays depicting a C1/2 fusion using C2 translaminar screws.

In general, the first procedure considered in the setting of AAI is a C1-2 fusion. This technique involves fusing axis to atlas, and several techniques exist. Gallie described the first technique in 1939 involving wiring and grafting [41]. Several authors have modified this technique [42–44]. The most commonly employed methods at this time involve C1/2 transarticular screw fixation or a combination of C1 lateral mass screws and C2 pars or pedicle screws. Magerl first described the use of C1-2 transarticular screws in 1986 [45, 46]. In this technique, 2 set screws are inserted through the C1-2 facets by a posterior approach. In addition, a midline bone graft may be inserted between C1 and C2 to provide a 3-point fixation [47]. Some surgeons may attempt to manually reduce the translational dislocation prior to fixation [40]. In 1994, Goel and Laheri proposed a plate and screw method for atlantoaxial fixation, which was later modified by Harms and Melcher in 2001, demonstrating the currently employed method of posterior C1 lateral mass screws and C2 pedicle or pars screws [48, 49]. In the Harms technique, polyaxial screws are inserted posteriorly into the lateral masses of C1 and into the pars of C2 bilaterally (Figure 7) [49]. If the pedicles of C2 are at least 6 mm wide, Alosh and colleagues suggest that pedicle screws may be placed. They demonstrated in a retrospective study of 93 patients and 170 screws that a pedicle diameter of less than 6 mm was associated with nearly a 2-fold increase in risk of cortical breach (37% versus 21%) [50]. It is also important for the surgeon to consider the course of the vertebral artery (VA) prior to C1/2 fusion. If the VA has an aberrant course, it may be unacceptably unsafe to place either C2 pedicle or C1/2 transarticular screws due to potential injury to the artery or violation of the foramen transversarium [51, 52]. C2 translaminar screws provide a safe alternative for fixation. These screws are inserted by placing a pilot hole at the junction of the spinous process and lamina along the cranial margin of the lamina. The angle of entry is kept in line with the slope of the lamina and the screw is inserted. The procedure is repeated along the caudal margin of the lamina on the contralateral side (Figure 8) [53].

**Figure 9:**
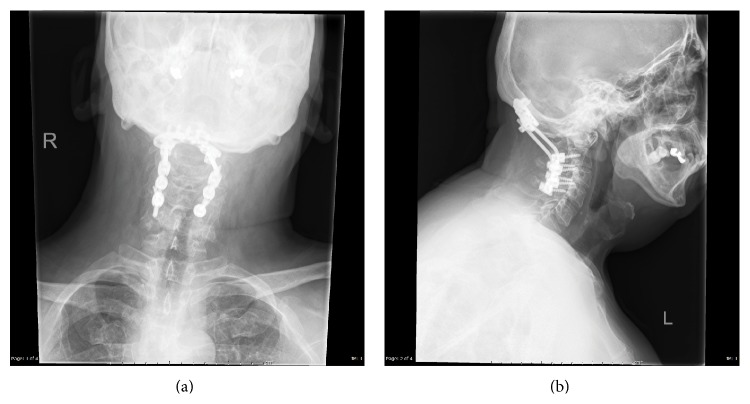
AP (a) and lateral (b) X-rays depicting an occipitocervical fusion.

In some cases, the AAI may occur posteriorly as well, resulting in dorsal compression and kinking of the spinal cord. In these cases, occipitocervical fusion may be employed [54]. In addition, this technique may also be preferred in cases in which AAS has progressed to cranial settling and vertical migration of the dens [55]. Grob first described the current technique used in 1991, which involves occipital plating and cervical screws (Figure 9) [47]. Furthermore, the dorsal compression due to a persistent pannus may necessitate a C1 laminectomy, in which case an occipitocervical fusion may be necessary.

As previously stated, if allowed to progress, AAI may result in CS, causing ventral compression of the cervicomedullary junction. The best surgical option in this case is odontoidectomy and ventral decompression, which can be performed through either microscopic transoral or endoscopic approach. The microscopic transoral approach for odontoidectomy involves retracting on the tongue and endotracheal tube to expose the odontoid via incision of the mucosa and pharyngeal musculature [56]. Neuronavigation may aid in maintaining midline orientation [3, 57]. The odontoid process is then drilled down and removed. Complications from this approach can include dysphonia, dysphagia, or minor CSF leak [57]. In some instances, postoperative airway obstruction and dysphagia may result in placement of tracheostomy and gastrostomy [58]. Because of these postoperative concerns, some surgeons prefer a transnasal or transoral endoscopic approach [57, 59, 60]. In a small series of 13 patients treated with endoscopic endonasal odontoidectomy, Yen et al. found that 85% (11/13) were extubated within 1 day of surgery [61]. Dasenbrock et al. found, in their cohort of 15 patients undergoing endoscopic image-guided transcervical odontoidectomy, that no patients required a postoperative tracheostomy [62]. Therefore, the endoscopic approach may achieve the same goals as the microscopic transoral route with less postoperative morbidity, which may be especially useful in the rheumatoid population in order to minimize operative risk.

SAS may develop as the first manifestation of RA in the cervical spine or as sequelae from prior fusion of a single level. For example, in a series of 33 patients undergoing surgery for AAI from RA, Clarke et al. found that 13 (39%) developed SAS after a C1-2 fusion [63], illustrating the risk of multisegment disease following fixation of a single level. Furthermore, Ito and colleagues demonstrated that, in patients undergoing C1-2 transarticular screw fixation for AAI, 57.6% (19/33) developed postoperative SAS [64]. In the treatment of SAS, the goals are to improve alignment of the subaxial cervical spine and decompress the spinal cord if necessary. These objectives can be accomplished via multilevel cervical laminectomy and fusion. The most commonly employed fusion technique involves placement of polyaxial, lateral mass screws connected by a rod. Roy-Camille, Magerl, An, and Anderson have each described variations of screw placement that differ by their entry point into the lateral mass and their trajectory [65–67]. The authors' preference is a modified Magerl technique with a slightly more superior and lateral trajectory in order to avoid injury to the exiting nerve root [68].

## 8. Surgical Outcomes in RA Patients

It is important to note that many of these RA patients are taking glucocorticoids (GC), DMARDs, and BAs, which may affect postsurgical outcomes. One of the established adverse effects of GC, particularly in doses of more than 5 mg daily, is a reduction of bone mineral density (BMD) leading to osteopenia and osteoporosis that can increase the risk of fracture [69, 70]. Decreased BMD is well known to adversely affect rates of fusion and hardware failure in spinal fusions at all levels and for a multitude of indications, including RA [3, 7]. This raises an obvious concern about the effects of GCs on the surgical outcomes of patients with cervical involvement of RA. In RA patients, bone loss occurs in GC naïve patients and may be a result of RA associated systemic inflammation, decreased weight-bearing activity from impaired mobility, decreased exposure to sunlight, and the fact that RA patients are predominantly postmenopausal women; all of which are well known risk factors for loss of BMD [70]. There have been recent trials that have demonstrated that DMARD therapy in combination with a low dose GC and osteoporosis prophylaxis, consisting of vitamin D and calcium supplementation in combination with a bisphosphonate, preserves BMD and in some instances results in an increase in BMD [69–72]. These findings should alleviate some of the concerns over the use of GC in regard to the effect of diminished BMD on surgical treatment of cervical disease. Additionally, a recent retrospective study of patients with RA undergoing spinal fusion at a single institution demonstrated that fusions at the craniovertebral junction could be safely performed on patients on long term GC, DMARD, and BA treatment [73]. The only notable difference in outcome was a smaller improvement in functional outcome in patients receiving higher doses of GC and BAs, which they attributed to those patients likely having more severe disease [73]. It is also important to note that patients with RA have a higher incidence of infection, particularly of bone, joints, skin, and soft tissues which can be partially attributed to the immunosuppressive effects of GCs, DMARDs, and BAs [3, 7, 69, 73, 74]. These agents have also been associated with an increased risk for serious postoperative infections [7, 75–78]. This predilection for infection should be weighed heavily when planning surgery for patients with cervical involvement of RA, consideration should be made for temporarily holding these medications, and these patients should be closely monitored for postoperative infectious complications [3, 7, 75, 76, 79]. In addition, as mentioned earlier, patients with severe cervical spine involvement tend to have lower limb disease as well [28, 29], resulting in a more challenging postoperative rehabilitation. This may affect the surgical objectives when taken into consideration. Lastly, patients with RA can have significant cardiovascular and pulmonary involvement that must be taken into consideration prior to any surgery in these patients. Strikingly, patients with RA have a similar risk of myocardial infarction as patients with diabetes mellitus or age 10 years their senior, underscoring the importance of thorough preoperative evaluation and medical optimization, which is well discussed in the literature, prior to any surgical procedure [75, 80].

Many studies have tracked the neurologic outcomes of patients undergoing surgery for cervical spine involvement of RA. As previously described, PADI may not only serve as a metric in preoperative evaluation but may also correlate to outcome. Boden et al. demonstrated that PADI might be the most important predictor of postoperative neurologic outcome in their case series of 73 patients [35]. The authors found that, in patients with paralysis due to AAI, no recovery occurred if the PADI was less than 10 mm, while recovery of at least one Ranawat class was achieved if the PADI was greater than 10 mm. Furthermore, if CS was found in addition to AAI, neurological recovery only occurred when the PADI was greater than 13 mm [35].

Preoperative Ranawat classification is also an important predictor of postoperative outcome. In a systematic review performed by Wolfs and colleagues, as expected, patients with a lower Ranawat classification preoperatively fared much better with respect to mortality, as 10-year survival rates were 77% and 63% for Ranawat I and II patients, respectively [81]. Additionally, survival analysis demonstrated that mortality rate was significantly worse for Ranawat IIIb patients. With respect to surgical outcome, 96% (182/752) of class I patients showed no deterioration in neurologic status, 53% (88/166) class II patients improved to grade I, 56% (125/223) of Ranawat IIIa patients improved 1 or 2 classes, and 21% (37/173) IIIb patients showed improvement by 1 or 2 classes [81]. This same study showed that conservative therapy did not improve Ranawat classification, and for those patients in class IIIa or IIIb, neurologic deterioration was inevitable [81]. In a case series by Nannapaneni and colleagues, 32 patients with Ranawat class IIIb myelopathy were treated for AAS by halo bracing, followed by posterior fusion with or without transoral decompression. In the 24 patients reaching final follow-up (median: 39 months) 14 were class IIIa and 4 were class II, while 6 remained class IIIb [82]. In another study, Matsunaga and colleagues found that 68% (13/19) of patients undergoing C1 laminectomy and occipitocervical fusion improved in Ranawat classification, while 76% (16/21) of patients treated conservatively showed neurologic deterioration [83]. In addition, as previously described, untreated AAI may have an impact on mortality. Tanaka et al. found that patients treated conservatively for AAI had a 1.7-fold increased mortality when compared to the treatment group (*N* = 26, 24; treatment, conservative, resp.) at 24-year follow-up [84].

## 9. Conclusion

The goal of current treatment strategies in RA is to prevent the involvement of the cervical spine. Patients with cervical spine involvement may present with neurological symptoms or headaches. RA involvement in the cervical spine may also present as an incidental finding in an asymptomatic patient. Through careful radiographic and clinical evaluation, patients with instability in the form of AAI, CS, or SAS can be detected. In patients that may require surgery, the operative risk must be weighed against the risk of conservative management, which, as previously mentioned, is not insignificant. The patient's pathology should dictate what surgery, if any, is performed. With careful patient selection using the above-mentioned parameters, surgery for RA in the cervical spine may not only promote neurologic recovery but also improve mortality.

## Figures and Tables

**Table 1:** Ranawat Classification of Rheumatoid Myelopathy [15].

Class I	Neurologically intact

Class II	Subjective weakness with hyperreflexia and dysesthesia

Class IIIa	Objective weakness with long tract signs but ambulatory

Class IIIb	Objective weakness with long tract signs with disability to walk or feed oneself, quadriparesis

**Table 2:** American Rheumatologic Association Classification of Global Functional Status [16].

Class I	Complete ability to carry on all usual duties without handicaps

Class II	Adequate for normal activities, despite handicap of discomfort or limited motion at one or more joints

Class III	Adequate for only few or none of the duties of usual occupation or self-care

Class IV	Incapacitated, largely or wholly bedridden, or confined to wheelchair, little or no self-care

**Table 3:** Indices of cranial settling [17].

	Description	Diagnostic criteria
Clark station	In the sagittal plane, divide the odontoid process into three equal parts, “stations,” and determine the level at which the anterior arch of C1 falls	Station I: anterior arch of C1 falls at the superior third (normal) Station II: anterior arch of C1 falls at middle third (mild) Station III: anterior arch of C1 falls at inferior third (severe)

Ranawat criterion	Distance between the center of the C2 pedicle and the transverse axis of C1 measured along the axis of the odontoid process	Measurements less than 15 mm (males) or 13 mm (females) are indicative of CS

Redlund-Johnell criterion	The distance between the inferior margin of C2 vertebral body and a line drawn from the posterior tip of the hard palate to the caudal cortical margin of the occiput (McGregor Line)	Measurements less than 34 mm (males) or 29 mm (females) are indicative of CS
